# Protocol for the development of a salutogenic intrapartum core outcome set (SIPCOS)

**DOI:** 10.1186/s12874-017-0341-5

**Published:** 2017-04-19

**Authors:** Valerie Smith, Deirdre Daly, Ingela Lundgren, Tine Eri, Cecily Begley, Mechthild M. Gross, Soo Downe, Zarko Alfirevic, Declan Devane

**Affiliations:** 1 School of Nursing and Midwifery, Aras Moyola, National University of Ireland, Galway, Ireland; 2 School of Nursing and Midwifery, Trinity College Dublin, 24 D’Olier Street, Dublin 2, Ireland; 3 Institute of Health and Care Sciences, The Sahlgrenska Academy, University of Gothenburg, Box 457, SE-405 30 Gothenburg, Sweden; 4 Faculty of Health Sciences, Department of Nursing and Health Promotion, Oslo and Akershus College, PO4 St. Olav Space, 0130 Oslo, Norway; 5 Midwifery Research and Education Unit, Hannover Medical School, Hannover, Niedersachsen Germany; 6 School of Community Health and Midwifery, Brook Building BB223, University of Central Lancashire, Preston, UK; 7 Women’s and Children’s Health, Institute of Translational Medicine, University of Liverpool, Crown St., Liverpool, L69 3BX UK

**Keywords:** Salutogenic, Salutogenesis, Core outcome set, COS, Intrapartum care, Maternity research, Maternity practice

## Abstract

**Background:**

Maternity intrapartum care research and clinical care more often focus on outcomes that minimise or prevent adverse health rather than on what constitutes positive health and wellbeing (salutogenesis). This was highlighted recently in a systematic review of reviews of intrapartum reported outcomes where only 8% of 1648 individual outcomes, from 102 systematic reviews, were agreed as being salutogenically-focused. Added to this is variation in the outcomes measured in individual studies rendering it very difficult for researchers to synthesise, fully, the evidence from studies on a particular topic. One of the suggested ways to address this is to develop and apply an agreed standardised set of outcomes, known as a ‘core outcome set’ (COS). In this paper we present a protocol for the development of a salutogenic intrapartum COS (SIPCOS) for use in maternity care research and a SIPCOS for measuring in daily intrapartum clinical care.

**Methods:**

The study proposes three phases in developing the final SIPCOSs. Phase one, which is complete, involved the conduct of a systematic review of reviews to identify a preliminary list of salutogenically-focused outcomes that had previously been reported in systematic reviews of intrapartum interventions. Sixteen unique salutogenically-focused outcome categories were identified. Phase two will involve prioritising these outcomes, from the perspective of key stakeholders (users of maternity services, clinicians and researchers) by asking them to rate the importance of each outcome for inclusion in the SIPCOSs. A final consensus meeting (phase three) will be held, bringing international stakeholders together to review the preliminary SIPCOSs resulting from the survey and to agree and finalise the final SIPCOSs for use in future maternity care research and daily clinical care.

**Discussion:**

The expectation in developing the SIPCOSs is that they will be collected and reported in all future studies evaluating intrapartum interventions and measured/recorded in future intrapartum clinical care, as routine, alongside other outcomes also deemed important in the context of the study or clinical scenario. Using the SIPCOSs in this way, will promote and encourage standardised measurements of positive health outcomes in maternity care, into the future.

## Background

Maternity care research and practice during labour more often focus on interventions that minimise or prevent adverse health outcomes [1]. Such a risk-reduction/avoidance approach can result in a limited understanding of, or an appreciation for, what constitutes positive health and wellbeing (salutogenesis) in intrapartum care. This was highlighted recently in a systematic review of systematic reviews, which was conducted by some authors of this paper [1]. The review, which was underpinned by Antonovsky’s theoretical framework of what constitutes salutogenesis [2], sought to identify salutogenically-focused reported outcomes in systematic reviews of intrapartum interventions. While the case for identifying salutogenically-focused outcomes is presented comprehensively in the published review that informs this protocol [1], in brief, the central argument is predicated on a need to move away from existing risk-avoidance/harm prevention approaches, to maternity care which has health promotion at its core. The review authors further suggest that an emphasis on risk in maternity care has led to increased routine interventions including caesarean section. In capturing only pathological outcomes, or ‘satisfaction’, studies fail to capture the positive added benefits of specific interventions, or lack of interventions. Consequently, understanding the nature and effects of salutogenic outcomes in maternity care is limited. The systematic review of reviews identified 136 (8%) salutogenically-focused outcomes only, from a total of 1648 reported outcomes across 102 intrapartum systematic reviews [1], further adding to the rationale for this study.

When trying to synthesise the evidence from studies on a particular topic, systematic reviewers are often faced with the difficulty of heterogeneity in the outcomes measured in those studies. One of the suggested ways to address this is to develop and apply agreed standardised sets of outcomes, known as ‘core outcome sets’ (COSs) [3–6]. The idea is that a COS should represent the minimum to be measured and reported in all trials, and other studies, on a specific condition, while accepting that outcomes outside of the COS might also be important in the context of the individual study [7]. Although a COS for evaluating maternity care exists, this was developed more-so for studies evaluating models of maternity care across the whole of the antenatal, intrapartum and postpartum periods [8]. A more focussed set, that specifically addresses salutogenic outcomes in intrapartum care, is needed. Using the outcomes identified in our systematic review of salutogenically-focused intrapartum outcomes, we propose to develop, through expert opinion and international consensus, a salutogenic intrapartum core outcome set (SIPCOS) for use in maternity care research and a SIPCOS for measuring and recording in daily intrapartum clinical care. The idea for the final maternity care research SIPCOS is to reflect all salutogenic outcomes that should be measured in all future studies, including systematic reviews, on intrapartum interventions, alongside condition-specific outcomes. For example, if researchers were developing a study on interventions for preventing post-partum haemorrhage (PPH), the selection of outcomes for measuring in the study may include a COS developed for studies on preventing PPH [9] alongside the outcomes from the SIPCOS, recognising that there may be some overlap of outcomes in the sets. Similarly, in developing an intrapartum-specific maternity care practice SIPCOS, the idea is for the SIPCOS to be measured and recorded on all women receiving intrapartum maternity care, as routine. In some instances, the SIPCOS and the condition-specific COS may be recording the same elements of care or outcomes, but they will be phrased differently (see Table 1 for examples). When this occurs, we would recommend that the SIPCOS phrasing is used. Using salutogenic COSs in this way, we believe, will promote and encourage the measurement of positive health outcomes, alongside other outcomes, in all intrapartum intervention studies and in clinical care, in the future.

**Table 1:** Salutogenically-focused outcomes identified by Smith et al. [1] and mapped to Downe et al. [10]

Smith et al. [1] Salutogenically-focused outcomes	Downe et al. [10] Positive pregnancy experience
Maternal satisfaction with care/experience	Positive labour and birth
Breastfeeding^a^ (e.g. initiation, duration, success)	
Control^a^ (perceived/personal control)	Autonomy
Maternal parenting confidence	Maternal self-esteem; Competence
Positive relationship with baby/bonding	Positive mothering
Wellbeing (mother/father; psychological/emotional)	Healthy pregnancy/healthy baby
Caregiver experience/satisfaction^a^	
Views^a^ (mother’s and/or father’s)	
Relaxation	
Mobile during labour	
Spontaneous Vaginal Birth	Normal birth
Pregnancy prolongation	
Spontaneous rupture of membranes	
Comfort	
Intact perineum	
Maternal perception of pain experienced^a^	

^a^positive reference

## Methods

### Aim

To present a protocol for developing salutogenic intrapartum core outcome sets (SIPCOSs) for use in maternity care research and for measuring and recording in daily maternity care practice.

### Design

The SIPCOS will be developed through international collaboration and perspectives. The development process will involve three phases.

#### Phase one

Phase one, which is complete, involved the conduct of the systematic review of reviews of intrapartum interventions to identify previously reported salutogenically-focused outcomes [1]. As an added measure to enhance comprehensiveness in this phase, we mapped the identified salutogenically-focused outcomes to the composite elements of a ‘positive pregnancy’ theme that emerged in a recent scoping review undertaken for the World Health Organisation (WHO), and focused on ‘what matters to women’ in terms of their pregnancy [10]. The outcomes were not described in exactly the same way as those identified by Smith et al. [1], but there were common underlying concepts; for example, *‘coping’* and ‘*parenting confidence’* reflected similarly *‘positive mothering’* and ‘*maternal self-esteem’* (see Table 1), and the concepts reported by Downe et al. [10] included all of the outcomes identified by Smith et al. [1]. The results of the systematic review of reviews, phase one of this study, provides an initial list of salutogenically-focused outcomes (Table 1) for use in phase two of the study. This initial list, as described, presents a preliminary set of outcomes proposed by the authors of the review that precedes the current study. There is a lack of multi-stakeholder involvement and agreement on whether these are the most important outcomes for including in a final SIPCOS. Phases two and three of the current proposed study, which include consensus methods, will develop an agreed SIPCOS that will be superior to an outcome set derived by a particular group of authors from a review of the literature alone.

#### Phase two

Phase two proposes an online, electronic Delphi survey, whereby participants in the survey will be asked to rate the importance of the outcomes identified in phase one, for inclusion in an intrapartum maternity care research SIPCOS and a daily intrapartum maternity care practice SIPCOS. Although the two SIPCOSs will be separate, they will be developed simultaneously during the survey process. The Delphi method is an optimum design for completing phase two of this proposed study as it will facilitate a means of consensus-building by using a series of questionnaires/data collection instruments to collect data from a panel of expert service users, clinicians, researchers and appropriate others on the topic under investigation [11]. Using an online approach will enable us to target wide international participation, albeit only English-speaking, at a relatively low cost. This method has been previously described and used for developing COSs in maternity care [8], and in other healthcare topics [12]. Launch of phase two, which will provide international consensus on a list of important salutogenically-focused outcomes for use in phase three, is planned for early 2017.

#### Phase three

Phase three, the final process in developing the SIPCOSs, will involve an international face-to-face consensus meeting, bringing together at least two members of each of the key stakeholder groups (users of maternity services, groups representative of users of maternity services, clinicians from all relevant disciplines, researchers, service funders, policy makers), from both low and medium/high income settings, to discuss, vote and agree on the final SIPCOSs for use in future maternity care research studies and for measuring and recording in daily intrapartum clinical care. Translators may need to be involved to facilitate the contributions from those in low income countries. Face-to-face meetings are used frequently in COS development processes as a means of facilitating discussion and equitable agreement on the final outcomes to be included in a COS [13].

## Details of methods

### Phase two: Online Delphi survey

#### Participants and recruitment

The target population for the online survey will be women as users of maternity services, individuals from groups representing users of maternity services, midwives, obstetricians, paediatricians, neonatologists, obstetric anaesthetists, doulas, maternity care researchers, service funders (including insurance companies, government funders, and private funders), and policy makers. These groups were selected to ensure wide stakeholder inclusion and involvement of all potentially relevant and interested parties. High, medium and low income countries will be represented. While there is no guidance that we are aware of on the optimum sample size for a Delphi consensus process, we propose to aim for 30 participants from each stakeholder group to ensure adequate representation, based on a sample size achieved in a previous COS development process [14], although we anticipate our numbers will likely be much greater. For the online survey, the target sample will be accessed through electronic discussion lists and professional organisations. Examples of these include, but are not limited to, international (i.e. in high, middle and low income countries such as UK, Australia, Canada, Czech Republic, Bulgaria, India, Zimbabwe) Colleges and Societies of Obstetricians and Gynaecologists, or equivalent groups, and international midwifery networks and associations, the Cochrane Pregnancy & Childbirth Reviewers’ Group, the Cochrane Pregnancy & Childbirth consumer networks, the International Confederation of Midwives network of research advisors and COST Action databases (e.g. COST Action IS1405: Building intrapartum research through health – an interdisciplinary whole system approach to understanding and contextualising physiological labour and birth). Purposeful sampling, to approach people with known expertise in maternity care, will be used. Snowball sampling will be used also, whereby participants from the above groups will be asked to forward the invitation to others whom they regard as having the required expertise. For listed groups targeting users of maternity services, in particular, we will recruit from these groups via the electronic discussion e-mail list manager. The manager (or chairperson of the group, details of which are publicly available on the listed groups’ websites) will be emailed with information on the survey and a request to distribute the invitation email to members on their email lists. The list managers will have an opportunity to contact us directly to clarify any issues or seek further information about the survey and the research prior to making a decision. The distribution of the survey, ultimately, will be at the discretion of the email list manager. There is precedent for survey distribution on matters related to maternity care by these groups (e.g. http://aimsireland.ie/what-matters-to-you-survey-2015/). An invitation e-mail will be circulated to potential participants via the electronic discussions lists (or via list managers where necessary) as above. Individuals who wish to participate will be requested to respond to the researcher with their name and personal e-mail address. On receipt of this the researcher will forward further information, instructions and the round 1 survey instrument, accessible only after formal consent is indicated by ticking the relevant box provided in the invitation email.

#### Data collection

A series of three sequential rounds to collect the survey data and condense the opinions of participants into group consensus (achieved on round 3) on what should comprise the minimum SIPCOS for use in intrapartum maternity care research and for measuring in daily practice, will be used. Responses to each round will be collated, analyzed, and redistributed to participants for further comment in successive rounds. Each round will have a response closing date 14 days after the date of invitation with a generic e-mail reminder sent on day 10 from the date of invitation. The number of participants responding to round 1 will be assessed and documented. The number of participants completing subsequent rounds will also be documented and attrition assessed. We will use an online survey software system to distribute the survey (e.g. www.surveymonkey.com).

##### Round 1

The first round instrument will contain a short questionnaire seeking participant demographic data and the rating instrument containing the salutogenically-focused outcomes identified in phase one, the systematic review of systematic reviews of intrapartum interventions (Table 1) [1]. The outcomes will be presented to participants for rating using a 9-point Likert scale as follows: 1–3 = not important, 4–6 = unsure of importance and 7–9 = important. Table 2 provides an example. To ensure completeness of outcomes, we will also invite participants, in this round, to add further ‘new’ outcomes (as ‘free-text’ option and not requested to score) that they would consider important or relevant for inclusion in the two SIPCOSs.

**Table 2:** Rating scale

Outcome	Not important			Unsure of importance			Important		
Breastfeeding○	1	2	3	4	5	6	7	8	9
For use in maternity care research	○	○	○	○	○	○	○	○	○
For use in daily maternity care practice	○	○	○	○	○	○	○	○	○

##### Round 2

In round 2, participants who responded to round 1 will be presented again with all of the outcomes after analysis of responses from round 1. Additional outcomes identified by participants in round 1 will be included in round 2. For each outcome from round 1, the rating results (percentages), for each outcome, from each group, will be presented. Participants will be asked to re-rate the importance of each outcome with knowledge of their, and the group’s, previous ratings. In addition, participants will be asked to rate the newly identified outcomes from round 1. All ratings will use the same Likert-type scale that was used in round 1.

##### Round 3

In round 3, participants who responded to round 2 will be presented with outcomes retained after analysis of responses from round 2. Each of the outcomes in the round 3 instrument will again be presented together with the rating percentages, for each outcome, from each group. In this round, participants, rather than rating each outcome on the scale, will be invited to answer two specific questions (Table 3):

1. Do you think this outcome is important for including in a SIPCOS for use in intrapartum (during labour and birth) maternity care research?
2. Do you think this outcome is important for including in a SIPCOS for use in daily intrapartum (during labour and birth) maternity care provision?

These questions form the basis for determining consensus as to whether or not an outcome will be included in the final SIPCOS. Inclusion will occur when ≥ 70% of members of at least three stakeholder groups (one of which must be users of care, as recommended in a previous COS development process [14]) consider the outcome as being important for including in the maternity care research COS or the maternity care practice COS.

**Table 3:** Round 3 instrument

	Do you think this outcome is important for including in a SIPCOS for use in		Do you think this outcome is important for including in a SIPCOS for use in	
	Intrapartum maternity care research		Intrapartum daily maternity care practice	
Outcome	Yes	No	Yes	No
Breastfeeding	O	O	O	O

#### Data analysis

All outcomes from round 1, including newly identified outcomes, will be forwarded to round 2. Outcomes achieving an ‘important’ rating (i.e. 7–9) of greater than or equal to 70%, in round 2, by any of the stakeholder groups, will be forwarded to round 3. In round 3, consensus on inclusion of an outcome in the preliminary maternity care research or daily practice SIPCOS will be determined based on ≥ 70% of all members of at least three stakeholder groups, *one of which must include users of maternity care,* responding ‘YES’ to the two questions posed in this round (Table 4 illustrates consensus). Valuing maternity care users in this way was informed by a previous COS development process which placed greater emphasis on users of healthcare in deciding what outcomes should be included in the final COS [14]. Outcomes that remain included in the preliminary SIPCOS at the end of the Delphi process will be brought forward to phase 3, the consensus meeting. Furthermore, the demographic details section of round 1 of the survey will capture information on the country of origin of participants. We therefore propose undertaking a sub-analysis of highly (very important) ranked outcomes by country to capture potential cultural variation in the ranking process.

**Table 4:** Preliminary consensus

Outcome	Proportion recommending inclusion of outcome in maternity care research SIPCOS					SIPCOS	
	User	MW	Ob	Paed/Neo	Research	Include in preliminary SIPCOS	Not to include in preliminary SIPCOS
Breastfeeding	90%	100%	70%	80%	70%	X	
Mobile during labour	55%	40%	30%	40%	30%		X

### Phase three: International face-to-face consensus meeting

Consensus on the final two SIPCOSs will be achieved through a face-to-face meeting of key stakeholders. The consensus meeting will bring together national and international representatives (translators may be required) from the key stakeholder groups to discuss, vote and agree on the final maternity care research and daily clinical care SIPCOSs. The consensus group will include, at a minimum, two representatives from each of the aforementioned stakeholder groups. The meeting is likely to include a brief presentation to participants on the preliminary SIPCOS development process, allocated time-frames for discussing the preliminary SIPCOS, dedicated time-frames for voting, including instructions on how to vote, and final agreement on the SIPCOSs. Outcomes that achieve an ‘include’ vote by ≥ 70% of the voting participants will be considered ‘consensus achieved’ and will be included in the final SIPCOSs for use in intrapartum maternity care research and in intrapartum maternity care daily practice.

#### Ethics

Ethical approval to conduct this study has been granted by Research Ethics Committee, National University of Ireland Galway, Ireland. Participation in the survey is by an ‘opt-in’ informed consent approach. Prior to accessing any of the survey items, participants will have received the study information leaflet (email) which contains the necessary information on which potential participants can base their decision as to whether or not they wish to participate in the survey. Participants will consent to participate by clicking on an ‘*I consent to participate in this study’* link prior to being able to access the round 1 instrument. The online survey software system used to facilitate the online survey maintains data behind a firewall. Only the researchers will have access to the data through use of a password and user identifier. To facilitate sending subsequent rounds to only those who participate in a previous round, participants will be requested to provide their email addresses. In this sense, the survey is not anonymous; however, all of the principles of data protection will still apply. Collated results only will appear in subsequent survey rounds and in publications arising from the study.

## Discussion

There is currently no salutogenic COS for studies evaluating intrapartum interventions or for measuring and recording outcomes in intrapartum clinical care. We propose developing such a COS (termed a SIPCOS) to improve synthesis of evidence in the future and to promote and encourage a standardised approach to measuring and recording positive health and wellbeing outcomes in women accessing intrapartum maternity care. We believe that using this SIPCOS will promote an appreciation for salutogenesis and encourage the measurement of positive health outcomes, alongside other outcomes, in the future. To ensure widespread awareness and use of the SIPCOS in research and in daily clinical care, the SIPCOS developed in this study will be disseminated widely to all participants in the survey, to known maternity care researchers, practitioners and users/groups representing users of maternity services, to the Cochrane Pregnancy and Childbirth Group for circulating to their members, to research funding bodies, to guideline/policy development groups and to initiatives such as COMET (Core Outcome Measures in Effectiveness Trials) for including in their COS database (http://www.comet-initiative.org/), CROWN (Core Outcomes in Women’s health, (www.crown-initiative.org) and WOMBAT (WOMen and Babies health and well-being: Action through Trials, (https://www.adelaide.edu.au/arch/research/res_network/WOMBAT/).

## Abbreviations

AIMS: Association for Improvements in Maternity Services
COS: Core outcome set
CROWN: Core outcomes in women’s health
SIPCOS: Salutogenic intrapartum core outcome set
WOMBAT: Women and babies health and well-being: action through trials
